# Epidemiology of injuries during Iran wheelchair basketball professional league: predictive risk factors and prevention strategies

**DOI:** 10.5249/jivr.v15i2.1846

**Published:** 2023-07

**Authors:** Mohammadreza Mahmoudkhani, Mehdi Norouzi, Zahra Fathi, Behnaz Charehjoo, Mona Oftadehgan, Fatemeh Alizadeh, Mahsa Miri

**Affiliations:** ^*a*^ Sport Medicine and Health Department, Faculty of Physical Education and Sport Sciences, University of Tehran, Tehran, Iran.

**Keywords:** Para-athletes, Athletic Injury, Epidemiology

## Abstract

**Background:**

Very few studies have investigated athletes with disabilities during a long period of competitions, such as a professional league. Also, there are limited findings related to specific mechanisms and risk factors of injury, and prevention strategies in Wheelchair Basketball. Therefore, the objective of this study was to investigate the rate and characteristics of injuries in the 2021-2022 Iran Wheelchair Basketball League and present prevention strategies.

**Methods:**

This retrospective study was conducted after the 2021-2022 (Mar 2021-Sep 2022) competition season. The sample size consists of 36 players, who were randomly selected among 129 players. All the data was processed using SPSS (version 21).

**Results:**

111 injuries were registered, equivalent to 132 per 100 players (95% CI: 100-180) and 8.16 Injuries per 1000 hours of athlete exposure (6.2-9.8). Also, 77.8% occurred during training and 22.2% in competitions. Most injuries affected the fingers and hands (35.13%), and shoulders (22.57%). The most common types of injuries were contusions (30.63%), laceration and skin lesion (23.42%), and muscle spasms (13.51%), in which, half of the injuries were slight (0-1 days), 27.8% (mild 4-7 days), and 22.2% moderate (8-28 days). Also, 66.9% of injuries were new, and 33.1% were recurrent. Most situations and actions leading to injury include quick wheelchair pushing (29.72%), the intense ball hitting (17.14%), and sudden stops or changes of direction of the wheelchair (12.63%). A multiple linear regression analysis (Enter method) demonstrated (R2 Adjusted=0.530) Wheelchair inappropriateness (P=0.015), lack of protective equipment (P=0.028), and previous injury (P=0.003) explained close to 55% of the injury rate.

**Conclusions:**

The injury rate during the league period was higher than the amounts reported from Paralympic games. Prevention strategies should be focused on rethinking athletes' pre-season readiness evaluation, return to play assessments and protection equipment technologies.

## Introduction

In recent decades, Wheelchair Basketball (WB) has become one of the most popular sports for people with disabilities.^1^ Functional classification rules in this sport allow a wide range of persons with different disabilities such as spinal cord injuries, lower limb amputations, polio, and other neuromuscular conditions to compete as a team.^2^ Basketball players are the second most injured athletes among wheelchair user’s athletes.^2^ Hence, research documents about the epidemiology of sports injuries and prevention strategies should be increased in this para-sport. Injury prevention investigations can help develop, implement, and evaluate injury prevention strategies by studying the incidence and severity of the injury, risk factors, and injury mechanisms.^3^ Some researchers investigated the epidemiology of injury in Wheelchair Basketball athletes during a short competition period such as a world championship (WC) and Paralympic games (PG).^3,4^ While, there is very limited research that has reported findings related to athletes with disabilities during a long period of competitions, such as a professional league. Also, there are limited findings related to specific mechanisms, injury risk factors, and prevention strategies in this popular para-sport. This study aimed to investigate the epidemiology of injury and identify predictive risk factors in the 2021-2022 Iran Men Wheelchair Basketball League (WBL) and present prevention strategies.

## Methods 

This retrospective study was conducted after the Iran Men Wheelchair Basketball League (WBL) 2021-2022 competition season (Mar 2021-Sep 2022). The sample size was calculated utilizing the G-Power 3.1 software, with a probability of 0.05 (minimum error type I), statistical power of 0.9 (minimum error type II), and effect size of 0.8 (mean effect). Thus, the minimum sample size was 20 participants. However, we registered 36 athletes representing 30 % of the total players. The study was conducted in accordance with the guidelines of the Declaration of Helsinki and was approved by the Research Ethics Committee of University of Tehran.

***Data collection***

The injury report form used in this study was adapted from forms used by the International Olympic Committee (IOC)^5^ with defined categories for the injury characteristics (anatomical region of injury, type, severity, mechanism, risk factor, and practice/competition). 

Three injury prevention specialists recorded injuries through interviews and by reviewing the full details of the injuries suffered - from the beginning of the season to the end of league matches – also, they asked participants to explain the specific action or situation that led to injury (as specific mechanisms of injury) and mention possible factors affecting injury (as specific risk factors) using the options proposed by the researchers. It should be noted, the injury was defined as any musculoskeletal problem or injury during training or competitions that required medical intervention, regardless of its consequences. Also, injury severity was categorized as slight (0–1 day), minimal (2–3 days), mild (4–7 days), moderate (8–28 days), and severe (>28 days).^6,7^

***Calculation of exposure and injury rate***

The athletes' exposure was determined based on the teams' training schedule and the number of matches. All the participants had 5 training sessions weekly, for 24 weeks, during the competition season. Athlete-match exposure was calculated by multiplying the number of players on the field by the number of games and athlete-training-day exposure as the number of players per team multiplied by the number of training sessions^8^ Athlete-days exposure was calculated by multiplying the number of players registered by the number of days of the league matches.^4^ The injury rates were calculated as the number of injuries per 100 players and per 1,000 athlete days and were reported with a confidence interval (CI) of 95%.^4^

The data's normality was confirmed using the Shapiro-Wilk test. Mean, standard deviation, and percentages were utilized to report data. The relative contributions of injury risk factors to injury rate were estimated using multiple linear regression analysis (Enter method). SPSS (version 21) was used to analyze the data.

## Results

The sample consists of 36 WB players, whose demographic characteristics are described in Table 1 . Table 2 shows the number of injuries, injury rate, and athletes’ exposure time.

**Table 1:** Characteristics of the participants.

Characteristics	Mean ±SD
Age (years)	29.67 ± 7.06
Seating height (cm)	94.2 ± 8.4
Weight (kg)	73.6 ± 9.3
Years of experience (years)	11.83 ± 5.86
Weekly practice (hours)	6.28 ± 2.74
Time of training session (hours)	2.39 ± 0.49
**Classification**	**Percentage**
1-1.5	47.2
2-2.5	25
3-3.5	5.6
4-4.5	22.2
**Type of disability**	**Percentage**
Spinal cord injury	38.9
Amputation	13.9
Polio	22.2
Physically disabled	22.2
Sensory motor	2.8

**Table 2:** Number of injuries, injury rate, athletes’ exposure time.

Number of	
Players	36
Training-days	4320
Match-days	27
All Injuries	111
Match injuries	28
Training injuries	83
**Injuries per 100 players (± CI 95%)**	
All injuries	132 (100-180)
Match injuries	33.3 (14.4-52.1)
Training injuries	116.6 (81.3-151.9)
**Injuries per 1,000 athlete-days (± CI 95%)**	
All injuries	25.2 (20.6-30)
Match injuries (Per 1,000 athlete-days of match)	44.4 (19.2-69.5)
Training injuries (Per 1,000 athlete-days of training)	9.7 (6.8-12.6)

One hundred and eleven injuries were documented as a whole, equal to 132 per 100 athletes [CI 95%: 100-180] or 25.2 per 1000 athlete-days [CI 95%: 20.6-30]. Also, 83 injuries occurred during training sessions, which equates to an incidence of 116.6 injuries per 100 players [CI 95%: 81.3-151.9] or 9.7 injuries per 1000 athlete-days of training [CI 95%: 6.8-12.6] and 28 injuries in match, equivalent to an incidence of 33.3 injuries per 100 players; [CI 95%: 14.4-52.1] or 44.4 injuries per 1000 athlete-days of training [CI 95%: 19.2- 69.5]. 

The most injured anatomical regions were fingers and hands (35.13%), shoulders (22.57%), and elbows (5.4%), respectively (Table 3).

**Table 3:** The most injured anatomical regions.

Anatomical region	number	Percentage
Hand & Fingers	39	35.13
Shoulder	25	22.57
Elbow	6	5.4
Arm	7	6.3
Forearm	5	4.5
Cervical Spain	4	3.6
Lumbar Spain	3	2.7
Pelvis	4	3.6
Wrist	5	4.5
Eye	3	2.7
Knee	7	6.3
Mouth & Tooth	3	2.7
Total	111	100

The most common types of injuries were contusions (30.63%), laceration and skin lesion (23.42%), and muscle spasms (Table 4) which, half of the injuries were slight (0-1 days), 27.8% (mild 4-7 days), and 22.2% moderate (8-28 days). Also, 66.9% of injuries were new and 33.1% were recurrent.

**Table 4:** The most common injury types.

Injury Types	Number	Percentage
Contusion	34	30.63
Laceration, Skin Lesion	26	23.42
Muscle Spasm	15	13.51
Strain	13	11.74
(Sub-)luxation/sprain	8	7.2
Tendinosis, Arthritis, or Similar	8	7.2
Blister	5	4.5
Impingement	2	1.8
Total	111	100

Most situations and actions leading to injury were included quick wheelchair pushing (29.72%), intense ball hitting (17.14%), sudden stops or changes of direction of the wheelchair (12.63%), direct contact with opponent’s wheelchair (13.51%) and direct contact with another player (8.1%) (Table 5). 

**Table 5:** The most common injury mechanisms.

Injury Mechanism	Number	Percentage
Quick Wheelchair Pushing	33	29.72
Intense Ball Hitting	19	17.14
Sudden Stops or Changes of Direction	14	12.63
Direct Contact with Opponent’s wheelchair	15	13.51
Direct Contact with Another Player	9	8.1
Fall on the Surface	7	6.3
Pivoting by Wheelchair	4	3.6
Attempt to Receive the Ball	7	6.3
Tight Strap	3	2.7
Total	111	100

The most common injury risk factors were Compact schedule of training and competitions (25.22%), wheelchair inappropriateness (20.72%), and Previous injury (18.95%) (Table 6).

**Table 6:** The most common injury risk factors.

injury risk factors	Number	Percentage
Compact schedule of training and competitions	28	25.22
Wheelchair inappropriateness	23	20.72
Previous injury	21	18.95
Technique and style of play of the opposing player	17	15.31
Insufficient physical fitness	8	7.2
Technical error	5	4.5
Non-standard flooring (high friction)	3	2.7
Lack of protection equipment	3	2.7
Technique and style of play of the opposing player	3	2.7
Total	111	100

Table 7 summarizes the indicators of the injury rate among Iran WBL players through injury risk factors. A multiple linear regression analysis (Enter method) demonstrated (R2 Adjusted=0.530) inadequate physical fitness (P=0.015), lack of protective equipment (P=0.028), and previous injury (P=0.003) explained close to 55% of the injury rate variance. 

**Table 7:** Multiple linear regression analysis to predict injury rate through injury risk factors.

Model 1	Unstandardized Coefficients (β)	Coefficients Std. Error	Standardized Coefficients (β)	T-Value	P-Value
Constant	0.888	0.412		2.155	0.040*
Technique and style of play of the opposing player	0.619	0.316	0.257	1.960	0.060
Insufficient physical fitness	0.717	0.276	0.327	2.594	0.015*
Technical error	0.015	0.517	0.005	0.030	0.976
Lack of protection equipment	1.297	0.557	0.289	2.327	0.028*
Wheelchair inappropriateness	0.416	0.293	0.202	1.422	0.167
Non-standard flooring (high friction)	0.092	0.488	0.026	0.189	0.851
Compact training and competitions schedule	0.518	0.387	0.178	1.338	0.192
Previous injury	0.955	0.294	0.394	3.251	0.003*
	**R=0.798**		**R2=0.637**		**R2 Adjusted=0.530**

a. Dependent Variable = Injury Rate * = P-Value ≤ 0.05

## Discussion

This is the first epidemiologic report of a professional WBL in which musculoskeletal injuries and predictive risk factors for injuries were investigated. The rate of injury was 83.7 injuries per 100 players or 12.12 injuries per 1,000 athlete days, and it was higher during competition than during the training sessions. Almost 60% of injuries occurred in the hand and shoulder. Just over half of the injury types were contusion and laceration and about one-third of injuries occurred during quick wheelchair pushing. Inadequate physical fitness (P=0.015), lack of protective equipment (P=0.028), and previous injury (P=0.003) explained close to 55% of the injury rate variances.

The injury rate among players who play in the Iran WBL was higher (25.5 injuries per 1,000 athlete-days [CI: 20.6-30]) than those reported during the 2012 PGs, with 12.0 (CI: 8.3-16.8) injuries per 1,000 athlete-days,^3^ and the 2016 PGs, with 12.8 (CI: 9.5-17.4) injuries per 1,000 athlete-days,^8^ and lower than the injury rate reported during the 2018 WBWC with 68.9 injuries per 1,000 athlete-days (CI: 55.4-82.4)4, and what was reported during the 2021 South America WBC with 94.5 injuries per 1,000 athlete-days (CI :75.3-113.7).^5^ Also, the training injury rate was lower than the matches injury rate which is consistent with other studies.^9^ Inconsistent findings could be explained by differences in the competition scheduling (uncompress competition schedule and the longer time interval between league matches) and its sensitivity and importance (international vs national). Also, inconsistent findings could result from the limited definition of injury in this study which emphasized medical intervention.

According to the biomechanics of wheelchair sports, it is expected that hand, wrist, and elbow suffered from more injuries due to involvement in all movements, technical demands, and tolerating repetitive stresses.^10^ In this study, most injuries occurred in the hands, shoulders, and elbows, respectively, which is consistent with the results of Bogado et al., (2023) but Hollander et al. (2020) and Gomez et al. (2017) reported the most injuries in the neck, back, and upper limbs, especially the shoulders.^3,4,10-12^ Inconsistent findings could be explained by the differences in competitions level and its technical demands which might cause more physical conflict in high-level matches, with hand-to-hand combats, close battles over possession of the ball, collisions with the opponent's wheelchair, and even fall and more risk of neck and back injuries.

It should note that the risk of shoulder injury is higher in people who use a wheelchair in their daily life than in people who use it only for games, and this risk is higher among wheelchair-users with a deficiency in trunk control.^13-15^ Thus maybe, WB players with trunk control deficiencies (players with 1 to 2.5 classification) need more recovery time compared to other players. Some studies have demonstrated they are at greater risk of fatigue.^16,17^ Moreover, coaches should consider longer recovery times for wheelchair user players than athletes who use it only for games. As the classification of athletes progresses toward severe disability, some considerations should be reviewed on an individual basis to reduce the risk of injury to people with more severe disabilities.

In the present study, the most common types of injuries were contusions (23.7%), scratches (13.2%), and muscle spasms (11.9%), respectively. Hollander et al. (2020) reported similar findings but they highlighted muscle spasms as the most common type. In addition, 52% of injuries were traumatic and, 48% of injuries were due to overuse mechanisms. Consistent findings have been reported during the 2012 PGs (65%)^3^ while overuse injuries predominated during the 2018 WBWC (52%)^4^ and 2021 SAWBC (53.8%) (Bogado et al., 2023). It seems that both mechanisms of injury (trauma and overuse) are challenging that need to be taken into account for WB injury prevention plans. 

Participants of this study reported most of the upper limb injuries occur when driving a wheelchair especially repetitive and rapid wheelchair pushing, and sudden deceleration or change of direction. Consistent with this finding, Hazmel et al. (2017) reported consistent findings.^10^ Prevention strategies have to be addressed the wheelchair pushing techniques in WB, regardless of competition level and technical proficiency, in both professional and amateur athletes. 

Also, to identify and correct the altered mechanics in wheelchair users with trunk control deficiencies, biomechanical assessments of the wheelchair pushing pattern should be considered in preventive actions in terms of ergonomic corrections and improvement of physical skills. Altered mechanics, is found to be a risk factor in this population.^13,14^

Our findings showed inadequate physical fitness, lack of protective equipment, and previous injuries positively associated with injury rate. The link between physical fitness and sports-related injuries has been documented through multiple studies.^18^ Also, Studies have shown that injury prevention programs improve different components of physical fitness as a modifiable intrinsic risk factor.^19,20^ Due to the diversity of Wheelchair Basketball players' disabilities, designing injury prevention programs should be considered with an emphasis on dynamic balance in the wheelchair and constant care of shoulder kinetics, especially for wheelchair users with trunk control deficiency. In addition, pre-season physical fitness assessment should be applied through prevention plans. 

Moreover, the lack of protective equipment was another indicator of the injury rate. WB is characterized by high-speed plays, sudden changes of direction, and frequent contact and collisions with other athletes. Hence, it could be assumed that part of the injury is related to the nature of the game. But it seems that the prevention strategies should be focused on rethinking protection devices technologies.^21^ For instance, wearable protection equipment could be added to athletes' suits.

Another interesting finding was that the previous injury was positively associated with the injury rate. Due to the changes along the kinematic chain, including proprioceptive deficits, restricted range of motion, excessive flexibility, and scar tissue formation, past injury has been considered the largest internal risk factor for future injury.^22^ There is evidence that when motor control is compromised, a past injury has a stronger influence on the likelihood of future injury. Without the proper intervention to restore motor control to its ideal state, the risk of future injury will rise.^23,24^ The likelihood of future injuries may be influenced by past injuries for brief periods of time, but with the right therapy, the association between past injuries and the likelihood of future injuries changes.^25^ Hence, prevention strategies should be focused on rethinking the rehabilitation programs and return-to-play assessments to evaluate the readiness of the affected body part in relation to returning to the WB.

## Conclusion

We concluded that the injury rate among WB players in Iran professional league was higher than those reported in the PGs but lower than those reported in the WBWC and SAWBC. Most of the injuries were seen on the hand/fingers and shoulder, a third was classified as contusions, and acute injuries constituted a little over half of all reported injuries. The majority of injuries were directly linked to the WB game technique, and one-third were related to wheelchair pushing. Inadequate physical fitness, lack of protective equipment, and previous injury were shown to be the greatest indicators of injury rate in WB players in professional league Iran.
